# Estimation of cardiac output variations induced by hemodynamic interventions using multi-beat analysis of arterial waveform: a comparative off-line study with transesophageal Doppler method during non-cardiac surgery

**DOI:** 10.1007/s10877-021-00679-z

**Published:** 2021-03-09

**Authors:** Arthur Le Gall, Fabrice Vallée, Jona Joachim, Alex Hong, Joaquim Matéo, Alexandre Mebazaa, Etienne Gayat

**Affiliations:** 1 Inria Paris-Saclay, 01, avenue Honoré d’Estienne d’Orves, 91120 Palaiseau, France; 2 LMS, École Polytechnique, 91128 Palaiseau Cedex, France; 3 Anesthesiology and Intensive Care Department, Lariboisière – Saint Louis – Fernand Widal University Hospitals, University of Paris, 02 rue Ambroise Paré, 75010 Paris, France; 4 UMR-S 942, INSERM, 02 rue Ambroise Paré, 75010 Paris, France; 5 Université de Paris, 85 boulevard Saint-Germain, 75006 Paris, France

**Keywords:** Cardiac output monitoring, Pulse contour analysis, Vasopressors, Fluid challenge, Trans-esophageal doppler, Multi-beat analysis of the radial pressure waveform

## Abstract

Multi-beat analysis (MBA) of the radial arterial pressure (AP) waveform is a new method that may improve cardiac output (CO) estimation via modelling of the confounding arterial wave reflection. We evaluated the precision and accuracy using the trending ability of the MBA method to estimate absolute CO and variations (ΔCO) during hemodynamic challenges. We reviewed the hemodynamic challenges (fluid challenge or vasopressors) performed when intra-operative hypotension occurred during non-cardiac surgery. The CO was calculated offline using transesophageal Doppler (TED) waveform (CO_TED_) or via application of the MBA algorithm onto the AP waveform (CO_MBA_) before and after hemodynamic challenges. We evaluated the precision and the accuracy according to the Bland & Altman method. We also assessed the trending ability of the MBA by evaluating the percentage of concordance with 15% exclusion zone between ΔCO_MBA_ and ΔCO_TED_. A non-inferiority margin was set at 87.5%. Among the 58 patients included, 23 (40%) received at least 1 fluid challenge, and 46 (81%) received at least 1 bolus of vasopressors. Before treatment, the CO_TED_ was 5.3 (IQR [4.1–8.1]) l min^−1^, and the CO_MBA_ was 4.1 (IQR [3–5.4]) l min^−1^. The agreement between CO_TED_ and CO_MBA_ was poor with a 70% percentage error. The bias and lower and upper limits of agreement between CO_TED_ and CO_MBA_ were 0.9 (CI_95_ = 0.82 to 1.07) l min^−1^, −2.8 (CI_95_ = −2.71 to−2.96) l min^−1^ and 4.7 (CI_95_ = 4.61 to 4.86) l min^−1^, respectively. After hemodynamic challenge, the percentage of concordance (PC) with 15% exclusion zone for ΔCO was 93 (CI_97.5_ = 90 to 97)%. In this retrospective offline analysis, the accuracy, limits of agreements and percentage error between TED and MBA for the absolute estimation of CO were poor, but the MBA could adequately track induced CO variations measured by TED. The MBA needs further evaluation in prospective studies to confirm those results in clinical practice conditions.

**Supplementary Information:**

The online version contains supplementary material available at 10.1007/s10877-021-00679-z.

## Introduction

Cardiac output (CO) monitoring is a cornerstone of the hemodynamic management during critical care or during high-risk surgical procedures. The ideal measurement technique should be accurate, precise, reactive and non-invasive to offer an optimal benefit/risk profile. However, none of the available techniques aggregate all of these characteristics.

Multi-beat analysis (MBA) of the arterial pressure waveform may improve the accuracy of CO estimation derived from non-calibrated arterial pressure waveform analysis by introducing a new method using biophysical mathematical modelling [1]. The MBA method was tested against intermittent thermodilution (ITD) with a pulmonary arterial catheter (PAC) in intra- and post-operative cardiac surgery settings [2, 3] and showed reasonable agreement. However, the question of the accuracy of the method to predict rapid CO variations (ΔCO) in response to hemodynamic challenges remains open.

Transesophageal Doppler (TED) is a minimally invasive method to assess CO. Although the TED method showed conflicting results for absolute CO measurements compared to ITD [4–6], it is very useful for the quantification of rapid changes in CO (ΔCO) caused by pathological (e.g., hemorrhage) or therapeutic (e.g., fluid or vasopressor challenges) phenomena [6–8].

TED is not an ideal method for absolute CO measurement, but our study evaluated the MBA method to estimate the absolute CO and the ΔCO caused by various therapeutic interventions during surgical procedures against the TED method in non-cardiac surgery.

## Methods

### Patients

This observational study was an offline retrospective analysis based on the data available in an electronic database that was prospectively constituted in our anesthesiology department from January 2014 to December 2016 in Lariboisière Hospital in Paris, France. The information related to the methodology of the database constitution is described in detailed elsewhere [9, 10]. Briefly, consecutive patients who were scheduled for neurosurgical or abdominal procedures were included in the database when continuous arterial pressure and cardiac output monitoring were mandatory for clinical purposes. Pregnant women and patients younger than 18 years old were not included. During the recording of the database, the patient's care was the responsibility of the senior anesthetist in charge in accordance with the protocols of our institution.

For the study purpose, we selected patients who were monitored using a fluid-filled radial arterial catheter (Plastimed, Prodimed, Saint-Leu-La-Forêt, France) and trans-esophageal Doppler (Deltex Medical, Chichester, UK), as indicated for clinical purposes.

### Ethical statement

Before the constitution of the prospective database, an appropriate Institutional Review Board (IRB), the ethics committee of the Société de Réanimation de Langue Française (CE-SRLF 11–356, 04th July 2013), approved our methodology and waived the requirement for written informed consent. Oral informed consent was obtained from all subjects after providing a protocol information letter. Every subject had the option to withdraw themselves at any time if they expressed their refusal.

According to a recent modification of the French law regarding data protection, another IRB examined and approved the retrospective analysis of the database (CERAR-SFAR 00,010,254-2020-007).

### Endpoints

The primary endpoint focused on the trending ability of the MBA method because the precision and accuracy evaluations of the MBA method using TED as the comparison method may represent a potential TED bias.

The primary endpoint was the concordance rate for relative ΔCO measurements between TED and MBA techniques after hemodynamic interventions.

The following secondary endpoints were used: (1) the bias and limits of agreements for absolute CO measurements and ΔCO values at baseline and during hemodynamic interventions; (2) the interchangeability rate, the percentage of error (PE), the coefficient of variation (CV) and the coefficient of error (CE) for CO estimation using MBA method at baseline; (3) the intraclass correlation coefficient at baseline and during hemodynamic interventions; and (4) the polar angle and radius describing the agreement between the two methods for ΔCO estimation.

### Doppler algorithm

We continuously recorded the Flow velocity waveform from which we calculated the stroke volume then the cardiac output. We first identified the systolic period of the considered heartbeats and calculated the velocity–time integral (VTI) of each heartbeat. The aortic diameter (AoD) was estimated using the formula given by Wolak et al. [11], in order to calculate the stroke volume (SV).

$$SV = \frac{3}{2} VTI \times \pi \left( \frac{AoD}{2} \right)^{2}$$

We finally calculated the CO by multiplying the SV by the heart rate.

### MBA algorithm

The MBA algorithm is currently commercially available in the US (Argos monitor, Retia Medical, Valhalla, NY, USA). However, our study is an offline retrospective analysis of hemodynamic signals that were recorded during the constitution of a prospective database of patients undergoing general anesthesia prior to the availability of the Argos monitor.

The classical pulse contour method for CO estimation is based on a beat-to-beat analysis of the arterial pressure waveform. Briefly, the systolic part of the arterial pressure waveform is transformed into CO using a transformation algorithm and weighted by a proportional factor (k) that aggregates the effect of arterial compliance, vascular tone, or other physiological phenomena [12, 13]. Many methods are used to determine this proportional factor k, but few methods allow for continuous estimation of this factor, which causes the vascular property to vary over time. The externally calibrated methods, which use intermittent thermodilution, allow adaptation and calibration of the CO estimation when changes in vascular properties occur. The pressure recording analytical method (PRAM) algorithm uses a deterministic model of the arterial pressure waveform and measures the area under the systolic and diastolic arterial waveforms in combination with the perturbation theory principle to estimate continuously the proportional change in volume related to change in pressure [14]. The method finally computes the CO using this modelled information. However, one of the limitations of the pulse contour methods is the presence of arterial wave reflections that may influence the accuracy of estimations of the vascular parameters.

The MBA method is not a beat-to-beat analysis of the arterial pressure waveform, but it allows a continuous estimation of vascular physiological properties. It uses a previously described long-term interval analysis (LTIA), which was validated against ITD [15]. It allows acquisition of rapid proportional changes in CO [16]. Briefly, an arterial pressure signal is simulated by the convolution of an impulse train model, calibrated by the measured cardiac rhythm and the measured pulse pressure, and an estimated impulse response function. The parameters of the impulse response function are adapted until the simulated arterial pressure best fits the measured arterial pressure signal. This impulse response function contains information on the vascular system because the exponential decay of this function may be fitted using a Windkessel model in a time-scale that is longer than heartbeats. Therefore, the time-constant parameter tau (τ = R * C, with R and C as the resistance and the compliance of the arterial system, respectively) is continuously estimated. The proportional change in CO in the LTIA method is estimated by the following formula:

$$\Delta CO \approx ~\frac{{\frac{1}{N}\mathop \sum \nolimits_{{t = 1}}^{N} ABP}}{\tau }$$

in which the upper term represents the mean arterial pressure over the chosen time window for the analysis, and N is the sampling of the arterial pressure waveform signal (ABP).

The MBA method includes an estimation of arterial compliance (C), which is calculated using a proprietary formula that involves patient-specific information (e.g., age, height, weight and gender) [16]. Therefore, using the Ohm’s law analogy, the absolute CO estimation is given by

$$CO = ~\frac{{\frac{1}{N}\mathop \sum \nolimits_{{t = 1}}^{N} ABP*C}}{\tau }.$$

### Data processing

We reviewed the anesthetic medical records in which the anesthetist in charge time-stamped hemodynamic interventions (vasopressors or fluid challenges) when they were requested. The vasopressors used in our anesthesiology department included 3 mg/ml ephedrine, 5 mcg/ml norepinephrine, and 50 mcg/ml phenylephrine. The choice and dose of the drugs was made at the physician’s discretion. Because the database constitution was non-interventional, the rate of administration, and the nature of the crystalloid used for fluid challenge was made at the physician’s discretion.

During general anesthesia, the TED and the arterial pressure devices were connected to a main monitor (Philips MP70, Philips, Einthoven, The Netherland). We extracted the signals using ixTrend software (Ixellence, Wildau, Germany), which allowed us to continuously gather the arterial pressure waveform and the aortic velocity waveform at a maximum sampling frequency of 125 Hz.

To perform a pulse contour analysis of arterial pressure recorded using a fluid-filled arterial catheter, a perfect pressure signal quality should be certified. The dampening of the system should be tested. Because the constitution of the database study was purely observational, we did not strictly control the hemodynamic procedures, but we firmly encouraged the anesthetists in charge to flush the arterial catheter. We analyzed only the arterial pressure signals that followed a fast-flush-test that met the quality criteria provided by Gardner et al. [17].

The Retia Algorithm is a proprietary algorithm, and the investigators did not have access to the transformation procedure from the arterial pressure waveform to CO_MBA_. An experienced engineer who was blinded to the CO_TED_ calculation applied the MBA algorithm to the arterial pressure waveform. We also analyzed the Doppler flow velocity waveforms from which we estimated the SV beat-to-beat (the descending aorta diameter was estimated using the formula provided in Wolak et al. [11]), and subsequently the CO_TED_.

Before comparative analysis of the two estimation methods, the Retia engineer and the university investigators reviewed all of the arterial pressure, TED waveforms, and the medical records containing the hemodynamic intervention time-stamps. We performed this review to ensure a perfect synchrony between the two estimation methods. It allowed us to verify the quality of the pressure signal (e.g., damping and stability) and the flow velocity waveform (e.g., diastolic flow, noise, and stability). We requested at least a 30 s of a stability period before signal analysis.

The hemodynamic interventions were grossly retrieved by using the correspondence between the time-stamped medical record and the raw PA and Doppler signals. The method was refined by identifying manually, on the PA signals, the fast-flush tests performed prior to the hemodynamic intervention. For each intervention, an artifact-free 30-s period before the intervention was identified. Two time periods were identified for the analysis; T1, corresponding to the baseline (within 30 s before the fast-flush test); and T2, corresponding to the time of maximal effect of the intervention. For fluid interventions, T2 was determined as a 30-s period around the maximum velocity–time integral, in a 15-min window after the initiation of the hemodynamic intervention. For vasopressor interventions, the window duration was shortened to 5 min, in which we searched for maximum MAP to account for the faster timescale associated with the effect of a vasopressor bolus compared to a fluid challenge.

The CO estimated from the two methods were averaged over the 30-s T1 and T2 periods. This resulted in 2 paired measurements for each intervention, which corresponded to CO_MBA_ and CO_TED_ before and after the intervention.

Examples of data selection are provided in supplementary materials Figures S1 and S2.

### Statistical analysis

For patient descriptions, continuous variables are represented as medians [Inter quartile range (IQR)]. Categorical variables are represented as numbers (percentage). We compared the CO_TED_ and the CO_MBA_ at baseline (T1) and their variations after therapeutic interventions (T2), and we performed a subgroup analysis of the nature of the hemodynamic intervention used (fluid or vasopressor challenges).

Considering an expected concordance rate of 96%, with an absolute difference of 8.5%, an alpha risk of 2.5% and a power of 90%, the number of subjects to include was 56 patients [18]. A non-inferiority margin was set to 87.5%. For the non-inferiority analysis, the results are provided with a 97.5% confidence interval (CI).

We followed recent methodological guidelines for validating new cardiac output estimation methods [19, 20]. Therefore, to address the accuracy and precision of the MBA method for absolute CO estimations, we (1) compared the weighted mean value of CO_TED_ with the weighted mean value of CO_MBA_ using weighted t-tests to consider repeated measurements within patients, and (2) provided a Bland & Altman plot and calculated the bias and the limits of agreements (LOA) for repeated measurements. We estimated the confidence intervals for the bias and the LOA. We also performed a meta-regression using the “METAFOR” package (R software, The R Foundation for Statistical Computing, Vienna, Austria) to consider the proportional bias, and a backward generalized linear multiple regression to identify the explicative variables associated with the proportional bias. (3) We calculated the percentage error (PE = 1.96*SD_MBA-TED_/Mean_TED_*100), the coefficient of variation (CV_MBA_ = SD_MBA_/Mean_MBA_), the coefficient of error (CE_MBA_ = CV_MBA_/sqrt(n_measurements_) and the precision (Prec_MBA_ = 2*CE_MBA_) of the MBA method. (4) We calculated the Intraclass correlation coefficient (ICC) for absolute agreements and consistency between the two methods using the “IRR” package of the R software. We considered the two-way mixed model because the two methods were performed for each measurement [21], and (5) we calculated the interchangeability rate according to Lorne et al. [22]. We first calculated the coefficient of repeatability (RC) of the Doppler method, considering a 48% PE for TED compared to ITD [23] (PE_ITD-TED_) and a 20% precision for ITD [24] (Prec_ITD_). We defined the limits of interchangeability as 1.96*CO_x_*CV_TED_, with CO_x_ being the continuous variable corresponding to the mean of the CO_TED_ and CO_MBA_, and CV_TED_ being the coefficient of variation of the Doppler method estimated by CV_TED_ = sqrt((PE_ITD-TED_)^2^ – (Prec_ITD_)^2^). We calculated the interchangeability rate as the number of the paired measurements within the interchangeability limits divided by the total number of paired measurements.

For the trending ability of the MBA method, we followed the aforementioned steps considering the absolute and the relative ΔCO. We additionally provided 4-quadrant plots and a polar plot and calculated the concordance rate with a 15% and 10% exclusion zone for 4 quadrants or polar plots, respectively, as recommended by Critchley et al. [25]. We performed the trending ability analysis in the different subgroups of interventions (fluids or vasopressors).

## Results

Between May 2014 and March 2017, 74 patients were screened for inclusion. Sixteen (22%) were not analyzed because of an inability to identify a clean Doppler or arterial pressure signal during the 30 consecutive seconds around the time-stamped baseline or peak challenge. Fifty-eight (78%) patients were included in the study. Thirty-nine (67%) patients were scheduled for neurosurgery, and 19 (32%) patients were scheduled for abdominal surgery. We analyzed 255 hemodynamic interventions. The median number of hemodynamic interventions by patient was 3 [1–6] (3 [1–6] for vasopressors and 2 [1, 2] for fluid challenges). The characteristics of the population are presented in Table 1. Twenty-three (40%) patients received at least 1 fluid challenge, and 46 (81%) patients received at least 1 administration of vasopressors.

**Table 1:** Population characteristics

	Population n = 58
Demography	
Age (years)	54 [43−63]
Women n(%)	25 (43)
Weight (kg)	70 [58−80]
Height (cm)	168 [163−175]
Body Mass Index	24 [20−27]
Comorbidities	
Hypertension n(%)	13 (26)
Diabete (%)	3 (6)
Dyslipidemia n(%)	4 (8)
Myocardial infarction n(%)	6 (12)
ASA	
I n(%)	10 (17)
II n (%)	42 (72)
III n(%)	6 (10)
Surgery	
Type of surgery	
Abdominal surgery n(%)	19 (32)
Neurosurgery n(%)	39 (67)
Length of surgery (min)	420 [390−540]
Pressors (number per patient)	3 [1−5]
Fluid (ml)	4250 [2125−7000]
Pressors (number of patients)	47 (81)
Fluid (number of patients)	23 (40)

Results are expressed as median [interquartile ranges] for continuous variables and number (%) for categorical variables

**Fig. 1:**
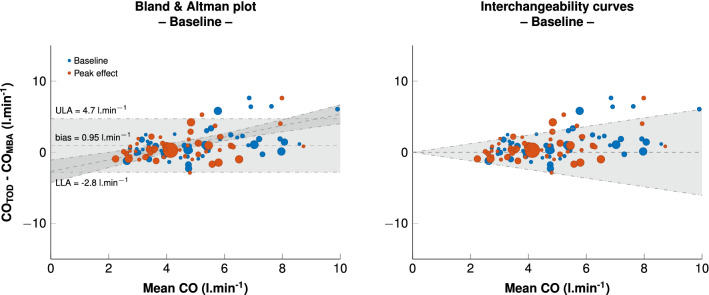
*Left* Bland and Altman plot for cardiac output assessment before (blue) and after (red) hemodynamic challenge between multi-beat analysis™(MBA) and Doppler (TED) methods. Data are represented as one dot per patient. The size of the dots represents the number of challenges per patient. Grey rectangles represent the confidence interval for the bias calculated for repeated measurements. A meta-regression was performed and is presented as a regression line with 95% confidence intervals. *Right* Interchangeability curve according to Lorne et al. [16]. The grey zone represents the interchangeability zone in which the two methods for CO estimation are considered interchangeable. Each dot represents one patient. The size of the dots represents the number of measurements performed in each patient. The interchangeability was achieved in 93% of the measurements

Before administration of any hemodynamic challenge, the median CO_TED_ across subjects was 5.3 (IQR [4.1–8.1]) l min^−1^, and the median CO_MBA_ was 4.1 (IQR [3–5.4]) l min^−1^ (p < 0.001). The key interchangeability indices are presented in Table 2. The Bland & Altman plot is shown in Fig. 1. The bias and lower and upper limits of agreement between CO_TED_ and CO_MBA_ were 0.9 (CI_95_ = 0.82 to 1.07) l min^−1^, −2.8 (CI_95_ = −2.71 to−2.96) l min^−1^ and 4.7 (CI_95_ = 4.61 to 4.86) l min^−1^, respectively. The interchangeability rate was 88%. As depicted in Fig. 1, a proportional bias was observed (p < 0.001). When analyzing the factors that influenced the bias, we observed that age, gender, weight, aorta diameter, and the duration and nature of the surgery (abdominal or neurosurgery) were independently associated with the bias (p < 0.05 for all). The PE for agreement between CO_TED_ and CO_MBA_ was 70%. The intraclass coefficient correlation (ICC) between CO_TED_ and CO_MBA_ at baseline was 0.45 (CI_95_ = 0.38 to 0.52; p = 0.005).

**Table 2:** Numerical results for concordance analysis

	All measurements	∆ Absolute	∆ Relative
Bland and Altman interpretation			
Bias [LLA–ULA]	0.9 [− 2.8–4.7] l min^−1^	−0.6 [− 2.7–1.5] l min^−1^	−1.8 [− 33–29] %
Coefficient variation, %	9	–	–
Coefficient error, %	2	–	–
Precision, %	4	–	–
Percentage error, %	70	–	–
Interchangeability rate, %	88	–	–
Concordance analysis			
Intraclass correlation coefficient (agreement)	0.4 [0.23–0.53]*	0.58 [0.34–0.72]*	0.77 [0.64–0.85]*
Pressors	–	0.5 [0.11–0.7]	0.67 [0.34–0.82]*
Fluids	–	0.67 [0.5–0.8]*	0.81 [0.69–0.88]*
Intraclass correlation coefficient (consistency)	0.45 [0.38–0.52]*	0.65 [0.57–0.72]*	0.8 [0.75–0.84]*
Pressors	–	0.61 [0.51–0.69]*	0.76 [0.69–0.81]*
Fluids	–	0.68 [0.5–0.81]*	0.81 [0.69–0.89]*
Percentage of concordance (°)	–	–	93 [90–97]
Pressors (°)	–	–	93 [89–97]
Fluids (°)	–	–	95 [79–100]
Proportional bias			
Linear regression (for 1 unit increase in CO_TOD_)	0.28 [0.1–0.47]* l min^−1^	0.43 [0.25–0.61]* l min^−1^	0.69 [0.35–1.03]* %
Polar description			
Polar angle (°)	–	− 15 [– 37 to 6]	–11 [– 35 to 14]
Pressors (°)	–	–16 [– 35 to 3.6]	–11 [– 31 to 10]
Fluids (°)	–	–9 [– 45 to 26]	–5 [– 43 to 34]
Length		– 1.04 [0.48–1.6] l min^−1^	25 [8–41] %
Pressors		– 0.98 [0.5–1.47] l min^−1^	21 [9–32] %
Fluids		– 1.07 [0.59–1.6] l min^−1^	29 [14–44] %

*LLA* lower limit of agreement, *ULA* upper limit of agreement, *CO*_*TED*_ cardiac output measured using Doppler method

*p < 0.001

**Fig. 2:**
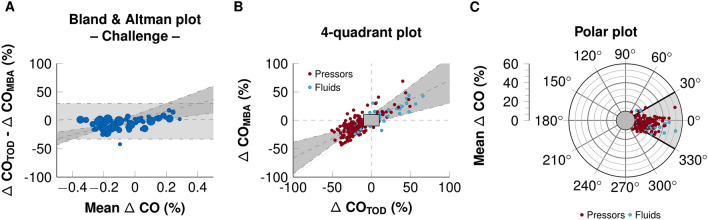
Concordance plots between the multi-beat analysis™ (MBA) method and Doppler (TED) method for relative cardiac output variation (ΔCO) assessment. **a** Bland and Altman plot for relative ΔCO in response to hemodynamic challenge. Data are represented as one blue dot per patient. The size of the dots represents the number of challenges per patient. Grey rectangles represent the confidence interval for the bias calculated for repeated measurements. A meta-regression was performed to visualize the proportional bias and is presented as a regression line with 95% confidence interval. **b** Four-quadrant plot for ΔCO in response to fluid challenge or vasopressor challenge. **c** Polar plot for ΔCO in response to fluid challenge or vasopressor challenge. Polar angles were calculated as the deviation with respect to the line of identity corresponding to 45°. The radius corresponds to the mean relative ΔCO measured using the two methods. Data are represented as one dot per cardiac output assessment

Based on the trending ability of the MBA method, the detailed results are shown in Table 2. When vasopressors were used, the CO_TED_ decreased by 1.4 ± 0.6 l min^−1^ and the CO_MBA_ decreased by 0.6 ± 0.4 l min^−1^ (p < 0.001). When fluids were used, the CO_TED_ increased by 0.45 ± 1.1 l min^−1^ and the CO_MBA_ increased by 0.3 ± 0.7 l min^−1^ (NS between ΔCO_TED_ and ΔCO_MBA_). The Bland & Altman plot for relative ΔCO is shown in Fig. 2a. When considering the absolute ΔCO, the nature of hemodynamic interventions (fluids or vasopressors) was also independently associated with the proportional bias, in addition to the factors identified at baseline (p < 0.05). In contrast, no association between the nature of intervention and bias was observed when considering the relative ΔCO. Therefore, we presented only the results of the relative ΔCO in the remaining document. The bias and lower and upper limits of agreement between relative ΔCO_TED_ and relative ΔCO_MBA_ were − 1.8 (CI_95_ = − 3.1 to − 0.6) % , − 33 (CI_95_ = − 35 to − 32)% and 29 (CI_95_ = 28 to 31)%, respectively. The Intraclass correlation coefficient between relative ΔCO_TED_ and ΔCO_MBA_ was 0.77 (CI_95_ = 0.64 to 0.85; p < 0.001) when considering all interventions, and 0.67 (CI_95_ = 0.34 to 0.82) and 0.81 (CI_95_ = 0.69 to 0.88), respectively, for vasopressors and fluids (p < 0.001 for both). The 4-quadrant plots and the polar plot are shown in Fig. 2b and c, respectively. We observed that the relative ΔCO measurements were close to the identity line, regardless of the hemodynamic challenge used. The percentage of concordance with the 15% exclusion zone between the relative ΔCO_TED_ and ΔCO_MBA_ was 93 (CI_97.5_ = 90 to 97)%, 95 (CI_97.5_ = 85 to 100)% and 93 (CI_97.5_ = 89 to 97)% when fluids and vasopressors were used, respectively. We also observed that 86 (CI_97.5_ = 81 to 91)% of the values were within the ± 30º polar angle with the 10% exclusion zones, 91 (CI_97.5_ = 79 to 100)% and 86 (CI_97.5_ = 80 to 91)% of the fluids and vasopressors, respectively.

## Discussion

The present study evaluated the ability of the MBA of the arterial pressure waveform method to estimate CO and accurately track the CO variations induced by hemodynamic interventions. Although the agreement with the Doppler method used was poor, we observed that the MBA method was not inferior to the TED method to estimate cardiac output variations caused by hemodynamic challenges, although vasopressors were used.

We first evaluated the precision and the accuracy of the MBA method for CO estimation compared with the TED method. We found a poor agreement, as demonstrated by the PE obtained with the Bland and Altman analysis. However, the interchangeability rate with TED was acceptable. One explanation may be the use of transesophageal Doppler as a method of comparison. In the two studies performed during and post-cardiac surgery, Saugel et al. [2] and Greiwe et al. [3] found substantially better results using intermittent thermodilution as a method of comparison (PE of 51 and 41%, respectively). Despite its excellent use to evaluate variations of CO [5], TED was less precise for absolute CO assessment [4]. A potential source of inaccuracy relies on the estimation of aorta diameter, which is necessary for the calculation of stroke volume from the measured flow velocity waveform. An 8% error in the diameter estimation led to a 16% error in CO measurement [26]. TED and MBA use a nomogram based on basic patient demographic information. In this retrospective and offline analysis, we did not use the built-in Deltex Medical nomogram that is meant to increase the accuracy of TED [26] with respect to ITD measurements because we recorded the raw flow velocity signals. Notably, our analysis showed that the aorta diameter was independently associated with the bias. Further studies are required to evaluate the ability of MBA to estimate absolute cardiac output in non-cardiac surgeries. Trans-cardiopulmonary intermittent thermodilution would be an interesting comparison method for precision and accuracy assessment.

Another source of inaccuracy was the nature of the surgery. The abdominal surgery in our hospital involves intraperitoneal chemotherapy-hyperthermy, which is associated with inflammatory and hyperdynamic states, but neurosurgical procedures are more often associated with restrictive behavior. These hyperdynamic states increased the observed bias between TED and MBA methods.

When considering our main objective of the trending ability of the MBA estimation method, the results obtained in the present study were consistent with the two clinical studies performed in peri-operative cardiac surgery, which validated the MBA method against intermittent thermodilution [2, 3]. The trending ability of the MBA technique in these two studies was tested by assessing CO at different time points during hemodynamic intervention. The concordance rates for CO measurement between MBA and ITD were 89% and 88%, respectively, when performed intra- [2] and post- [3] operatively.

One interesting finding of our study was the ability of MBA to track CO during vasopressor-induced variations. Most of the non-externally calibrated CO estimation methods using arterial pressure waveform analysis are misled when vasopressors are used [12, 27–29]. Although all of these techniques share a physical principle, their ability (or their difficulty) to accurately estimate the vascular physiology introduces a source of bias. During vasopressor administration, the vascular properties are modified, and the cardiac output response may vary despite the increased arterial pressure response is consistent. Depending on the patient’s volemia status prior to vasopressor administration, the vascular tone variation has various effects on the net effect, and the response may be an increase or a decrease in CO [30, 31].

Based on the presented results, and even if the results related to vasopressors seem potentially relevant, the MBA cannot be recommended for routine CO monitoring. Further studies are required to address this issue prospectively.

We also analyzed the bias between ΔCO_TED_ and ΔCO_MBA._ The nature of the hemodynamic interventions was associated with the proportional bias. The fluid challenge increased cardiac output. However, vasopressors were mostly associated with a decrease in cardiac output in our study. The magnitude of change in cardiac output affected the difference between ΔCO_TED_ and ΔCO_MBA_, which resulted in a proportional bias. We observed that the vasopressor-induced decrease in CO was higher than the fluid challenge-induced increase in CO. Because the relative changes allowed us to overcome this bias, the statistical association was not observed for the relative ΔCO.

The main limitation of our study relies on the use of a non-gold standard reference method for cardiac output measurement. Although thermodilution is the reference method for measuring cardiac output and its variations over time, this technique may not be convenient for use in routine clinical practice in anesthesia for cerebral and abdominal surgery. In this study we used TED as the reference method because of its minimally invasive, easy to use and acceptable trending ability for induced CO variations by hemodynamic challenges [6–8]. Therefore, the flow velocity signal was continuously recorded. We calculated the stroke volume then the cardiac output, by using an algorithm based on estimation of aorta diameter, the latter being associated with the observed bias between CO _TED_ and CO_MBA_. Therefore, the results of the Bland & Altman plot should be taken with caution. This work is exploratory and the ability of the MBA to measure reliable absolute values of CO remains to be tested using reference method such as thermodilution.

However, despite very poor agreements between the two methods, the trending ability of the MBA method was substantially better, when compared with TED method. The TED method is not a gold standard reference method for absolute CO measurement and showed conflicting results in various publications [4, 5]. However, its ability and reactivity to measure trends in CO are interesting characteristics to evaluate rapid modifications of the hemodynamic state that may be induced during therapeutic interventions (e.g. fluid challenge, vasopressor infusion, and passive leg raising) [5]. We think that this result showing a poor agreement but a good trending may still be seen as a promising result. Indeed, if we can understand the limitations of static TED values as a reference method for CO measurement, we can reasonably believe that TED trending is adequate because the variations in measured blood velocities in the descending aorta should reflect the real variations in cardiac output during therapeutic intervention [5]. The MBA algorithm continuously estimates vascular parameters (diastolic time constant τ), which aggregates compliance and resistance of the vascular system, all of which would be reasonably affected when using vasopressors. The good trending performances observed when comparing CO_TED_ and CO_MBA_ variations can therefore be considered as an encouraging result for MBA since this pulse contour algorithm would adequately reflect the variations in cardiac output induced especially by vasopressors. We believe that this result is interesting and has to be confirmed in further studies.

Several factors limited the internal and external validity of our study. First, the retrospective and offline design of our analysis is a limitation. We did not use the value given by the Deltex monitor for CO assessment but rather the Doppler flow velocity waveform, converted into CO via calculation. The absolute reference value for CO may be biased due to this calculation. However, the proportional change in CO remains valid because the source of inaccuracy relied on the aorta diameter estimation, which was fixed in the Deltex algorithm.

Second, the signal processing may have introduced bias. To prevent the algorithms for CO calculation from being biased by the arterial pressure or Doppler velocity waveform artifacts, we carefully selected the analyzed periods. We selected the time period for fluid and vasopressor administration by hand and applied the algorithm for CO estimation on the arterial pressure waveform for MBA method and the Doppler velocity waveform for TED method, respectively. The manual selection of the time periods may have affected the CO calculation. However, two experienced engineers were in charge of the data post-processing procedure. The two engineers simultaneously performed the time selection. One of the engineers calculated the CO_MBA_ from the arterial pressure waveform, and the other engineer calculated the CO_TED_ from the Doppler velocity waveform. Each investigator was blinded to the results of the other investigator.

Another limitation is the number of patients included, which was small for the duration of the recording (less than two patients per month). It introduces a selection bias. This small sample may be explained by the difficulty in obtaining a good quality signal for algorithm application. This method will be improved with the use of the built-in commercial algorithm that is included in dedicated monitors.

We also observed that the subgroup analysis lacked power to conclude of the non-inferiority of the MBA technique to estimate ΔCO in response to fluid challenges.

## Conclusion

In this off-line analysis, MBA demonstrated poor accuracy, limits of agreement and percentage error with respect to TED for absolute estimation of CO. However, it demonstrated good performance in estimating the CO variations during hemodynamic challenges compared to TED, even when vasopressors were used. The absolute CO estimation in non-cardiac surgery should be tested in a prospective study using a reference method comparison with the commercially available Argos monitor.

## Supplementary Information

Below is the link to the electronic supplementary material.

Supplementary Information FigureS1. Bland & Altman plot for absolute CO in response to hemodynamic challenge. Data are represented as one blue dot per patient. The size of the dots represents the number of challenges per patient. Grey rectangles represent the confidence interval for the bias calculated for repeated measurements. A meta-regression was performed to visualize the proportional bias and is presented as a regression line with 95% confidence interval (PDF 105 kb)

Supplementary Information FigureS2. Example of signal analysis for vasopressor administration. The administration of the vasopressor follows the fast-flush test. The T1 - baseline period corresponds to the 30 seconds following the fast-flush test. The T2 -peak period corresponds to the period of maximal pressure following vasopressor administration. In blue the arterial pressure signal, in red the Blood flow velocity signal measured by trans-esophageal Doppler. Example of signal analysis for fluid administration. The administration of the fluid follows the fast-flush test. The T1 - baseline period corresponds to the 30 seconds following the fast-flush test. The T2 -peak period corresponds to the period of maximal velocity time integral following fluid administration. In blue the arterial pressure signal, in red the Blood flow velocity signal measured by trans-esophageal Doppler (PDF 71 kb)

## Data Availability

The data will be rendered available on demand to the corresponding author.

## References

[1] Retia Medical LLC. *Argos Cardiac Output Monitor Operator’s Manual*. Retrieved 18 Sep 2020 from https://www.retiamedical.com/wp/wp-content/uploads/2020/10/60-001-Rev-C-Appendix-A-Argos-Cardiac-Output-Monitor-Operators-Manual.pdf.

[2] Saugel B, Heeschen J, Hapfelmeier A, Romagnoli S, Greiwe G (2019). Cardiac output estimation using multi-beat analysis of the radial arterial blood pressure waveform: a method comparison study in patients having off-pump coronary artery bypass surgery using intermittent pulmonary artery thermodilution as the reference method. J Clin Monit Comput..

[3] Greiwe G, Peters V, Hapfelmeier A, Romagnoli S, Kubik M, Saugel B (2019). Cardiac output estimation by multi-beat analysis of the radial arterial blood pressure waveform versus intermittent pulmonary artery thermodilution: a method comparison study in patients treated in the intensive care unit after off-pump coronary artery bypass surgery. J Clin Monit Comput..

[4] Peyton PJ, Chong SW (2010). Minimally invasive measurement of cardiac output during surgery and critical care: a meta-analysis of accuracy and precision. Anesthesiology.

[5] Dark PM, Singer M (2004). The validity of trans-esophageal Doppler ultrasonography as a measure of cardiac output in critically ill adults. Intensive Care Med..

[6] Møller-Sørensen H, Cordtz J, Østergaard M, Nilsson JC, Hansen KL (2017). Transesophageal Doppler reliably tracks changes in cardiac output in comparison with intermittent pulmonary artery thermodilution in cardiac surgery patients. J Clin Monit Comput..

[7] Chytra I, Pradl R, Bosman R, Pelnář P, Kasal E, Židková A (2007). Esophageal Doppler-guided fluid management decreases blood lactate levels in multiple-trauma patients: a randomized controlled trial. Crit Care.

[8] Phan TD, Ismail H, Heriot AG, Ho KM (2008). Improving perioperative outcomes: fluid optimization with the esophageal doppler monitor, a metaanalysis and review. J Am Coll Surg..

[9] Vallée F, Passouant O, Le Gall A, Joachim J, Mateo J, Mebazaa A (2017). Norepinephrine reduces arterial compliance less than phenylephrine when treating general anesthesia-induced arterial hypotension. Acta Anaesthesiol Scand..

[10] Vallée F, Le Gall A, Joachim J, Passouant O, Matéo J, Mari A (2017). Beat-by-beat assessment of cardiac afterload using descending aortic velocity–pressure loop during general anesthesia: a pilot study. J Clin Monit Comput..

[11] Wolak A, Gransar H, Thomson LEJ, Friedman JD, Hachamovitch R, Gutstein A (2008). Aortic size assessment by noncontrast cardiac computed tomography: normal limits by age, gender, and body surface area. JACC Cardiovasc Imaging.

[12] Caillard A, Gayat E, Tantot A, Dubreuil G, M’Bakulu E, Madadaki C (2015). Comparison of cardiac output measured by oesophageal Doppler ultrasonography or pulse pressure contour wave analysis. Br J Anaesth..

[13] Mehta Y (2014). Newer methods of cardiac output monitoring. World J Cardiol..

[14] Giomarelli P (2004). Cardiac output monitoring by pressure recording analytical method in cardiac surgery. Eur J Cardiothorac Surg..

[15] Zhang G, Mukkamala R (2012). Continuous and minimally invasive cardiac output monitoring by long time interval analysis of a radial arterial pressure waveform: assessment using a large, public intensive care unit patient database. Br J Anaesth..

[16] Mukkamala R, Reisner AT, Hojman HM, Mark RG, Cohen RJ (2006). Continuous cardiac output monitoring by peripheral blood pressure waveform analysis. IEEE Trans Biomed Eng..

[17] Gardner RM (1981). Direct blood pressure measurement–dynamic response requirements. Anesthesiology.

[18] Glueck DH (2008). Sample size calculations in clinical research 2nd edition by CHOW, S.-C., SHAO, J., and WANG, H. Biometrics.

[19] Montenij LJ, Buhre WF, Jansen JR, Kruitwagen CL, de Waal EE (2016). Methodology of method comparison studies evaluating the validity of cardiac output monitors: a stepwise approach and checklist † †This Article is accompanied by Editorial Aew110. Br J Anaesth.

[20] Odor PM, Bampoe S, Cecconi M (2017). Cardiac output monitoring: validation studies–how results should be presented. Curr Anesthesiol Rep..

[21] Koo TK, Li MY (2016). A guideline of selecting and reporting intraclass correlation coefficients for reliability research. J Chiropr Med..

[22] Lorne E, Diouf M, de Wilde RBP, Fischer M-O (2018). Assessment of interchangeability rate between 2 methods of measurements: an example with a cardiac output comparison study. Medicine (Baltimore).

[23] Critchley LA, Critchley JA (1999). A meta-analysis of studies using bias and precision statistics to compare cardiac output measurement techniques. J Clin Monit Comput..

[24] Cecconi M, Rhodes A, Poloniecki J, Della Rocca G, Grounds RM (2009). Bench-to-bedside review: the importance of the precision of the reference technique in method comparison studies–with specific reference to the measurement of cardiac output. Crit Care.

[25] Critchley LA, Lee A, Ho AM (2010). A critical review of the ability of continuous cardiac output monitors to measure trends in cardiac output. Anesth Analg..

[26] Singer M (2009). Oesophageal Doppler. Curr Opin Crit Care.

[27] Monnet X, Anguel N, Naudin B, Jabot J, Richard C, Teboul J-L (2010). Arterial pressure-based cardiac output in septic patients: different accuracy of pulse contour and uncalibrated pressure waveform devices. Crit Care Lond Engl..

[28] Metzelder S, Coburn M, Fries M, Reinges M, Reich S, Rossaint R (2011). Performance of cardiac output measurement derived from arterial pressure waveform analysis in patients requiring high-dose vasopressor therapy. Br J Anaesth..

[29] Slagt C, Malagon I, Groeneveld ABJ (2014). Systematic review of uncalibrated arterial pressure waveform analysis to determine cardiac output and stroke volume variation. Br J Anaesth..

[30] Cannesson M, Jian Z, Chen G, Vu TQ, Hatib F (2012). Effects of phenylephrine on cardiac output and venous return depend on the position of the heart on the Frank-Starling relationship. J Appl Physiol..

[31] Maas JJ, Pinsky MR, de Wilde RB, de Jonge E, Jansen JR (2013). Cardiac output response to norepinephrine in postoperative cardiac surgery patients: interpretation with venous return and cardiac function curves. Crit Care Med..

