# Supplementary figures and images for: Estimation of cardiac output variations induced by hemodynamic interventions using multi-beat analysis of arterial waveform: a comparative off-line study with transesophageal Doppler method during non-cardiac surgery
Source: J Clin Monit Comput. 2021 Mar 9;36(2):501–10. doi: 10.1007/s10877-021-00679-z (PMC9123019; doi:10.1007/s10877-021-00679-z)

### Arterial Blood Pressure

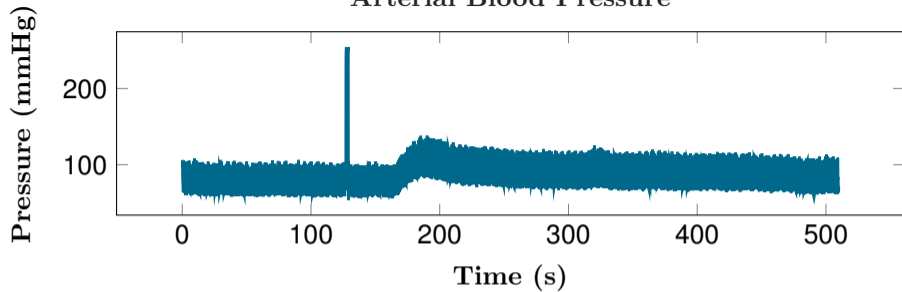

### Aortic Blood Velocity

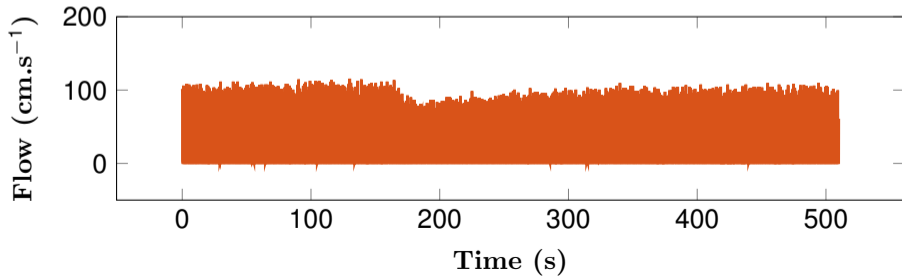

Supplement: Supplementary file 1 — Supplementary Information FigureS1. Bland & Altman plot for absolute CO in response to hemodynamic challenge. Data are represented as one blue dot per patient. The size of the dots represents the number of challenges per patient. Grey rectangles represent the confidence interval for the bias calculated for repeated measurements. A meta-regression was performed to visualize the proportional bias and is presented as a regression line with 95% confidence interval (PDF 105 kb) [file 10877_2021_679_MOESM1_ESM.pdf]

### Arterial Blood Pressure

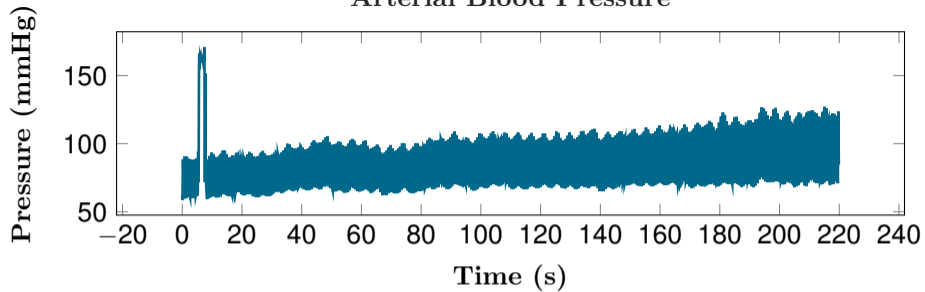

### Aortic Blood Velocity

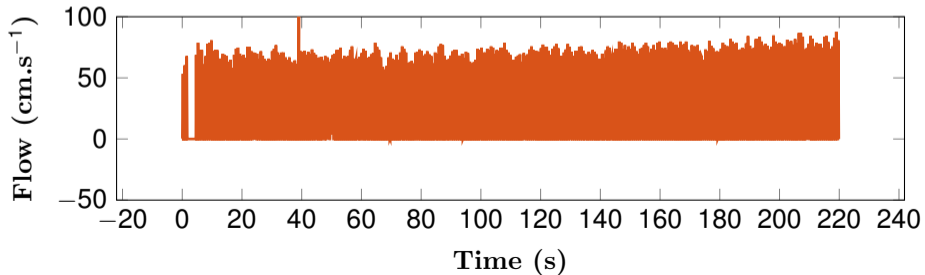

Supplement: Supplementary file 2 — Supplementary Information FigureS2. Example of signal analysis for vasopressor administration. The administration of the vasopressor follows the fast-flush test. The T1 - baseline period corresponds to the 30 seconds following the fast-flush test. The T2 -peak period corresponds to the period of maximal pressure following vasopressor administration. In blue the arterial pressure signal, in red the Blood flow velocity signal measured by trans-esophageal Doppler. Example of signal analysis for fluid administration. The administration of the fluid follows the fast-flush test. The T1 - baseline period corresponds to the 30 seconds following the fast-flush test. The T2 -peak period corresponds to the period of maximal velocity time integral following fluid administration. In blue the arterial pressure signal, in red the Blood flow velocity signal measured by trans-esophageal Doppler (PDF 71 kb) [file 10877_2021_679_MOESM2_ESM.pdf]
